# Clinical significance of golgi protein‐73 as a diagnostic marker for Egyptian patients with colorectal cancer: Preliminary study

**DOI:** 10.1002/cnr2.1379

**Published:** 2021-05-20

**Authors:** Shaymaa Abdelraheem Abdelhady, Heba Attya, Mohamed Abdo, Fadia M. Attia

**Affiliations:** ^1^ Department of Clinical Pathology Suez Canal University Ismailia Egypt; ^2^ Department of Family Medicine Suez Canal University Ismailia Egypt; ^3^ Department of Internal Medicine Suez Canal University Ismailia Egypt

**Keywords:** colorectal cancer, diagnostic biomarker, golgi membrane protein (GP73)

## Abstract

**Background:**

Colorectal cancer is one of the most common cancers and the leading cause of cancer‐related death worldwide. Early diagnostic methods help in therapeutic success and higher survival rate. Golgi protein 73 (gp73) could help in diagnosis of colorectal cancer at an earlier stage.

**Aim:**

A case‐control study aimed to assess serum level of golgi protein 73 (gp73) as a liquid biopsy marker in Egyptian colorectal cancer patients.

**Methods and results:**

In the current study, ninty (90) patients were included and classified into three groups; thirty (30) patients with Colorectal cancer (CRC) as study group; 30 patients (20 patients with irritable bowel disease and 10 patients with rectal polyps) as pathological control and 30 healthy adult individuals as normal control. The diagnosis was based on the history, clinical, laboratory, endoscopic, and histological data. Golgiprotein 73 (GP73) was measured by ELISA immunoassay Kit. Serum GP73 level was higher in CRC patients than pathological control group and normal control group with high sensitivity and specificity *p* < .005.

**Conclusion:**

GP73 alone or combined with Carcinoembryonic antigen (CEA) may be good diagnositic marker in CRC. However large studies are warranted on different stages of the disease to assess its diagnostic and prognositic value.

## 1 INTRODUCTION

Colorectal cancer (CRC) is characterized by accumulation of environmental factors, genetic mutations, and epigenetic changes in the colonic epithelium that eventually results in neoplastic transformation.^1^

Various screening and diagnostic methods for CRC are available, the methods range from invasive and costly procedures such as colonoscopy to cheap and noninvasive tests such as the fecal occult blood test.^2^ While colonoscopy and sigmoidoscopy are the most sensitive procedures for diagnostic examinations, these procedures are difficult to implement on a population‐wide basis due to many disadvantages including cost, invasiveness, a higher risk of perforation, and postprocedural bleeding.^3^ On the other hand, the cheap and noninvasive fecal occult blood test has poor patient compliance, variations in analytical procedures such as different methods of stool collection and handling, the need for multiple test samples, and variations in the interpretation of test.^4^

Golgi protein 73 (GP73) is a 73‐kD transmembrane glycoprotein that located in the cis‐Golgi complex.^5^ It is also known as Golgi membrane protein 1 (GOLM1) or Golgi phosphoprotein 2 is expressed primarily in epithelial cells which is highly expressed in the colon, stomach, prostate, and trachea in normal healthy persons..^6^

Golgi has been shown to play an active role in cell migration through posttranslational modification and prominent changes in the Golgi apparatus, as evidenced by the disruption of biochemical composition, structure, and functional levels observed in human carcinogenesis and metastasis.^7^ Gp73 is also expressed colon cancer, a finding that may have diagnostic value.^8^ So, it is important to assess the role of GP73 as a diagnostic tumor marker in patients with CRC.

## 2 SUBJECTS AND METHODS

This case–control study was carried out at Suez Canal University hospital, Egypt in the period from June 2019 to June 2020. The study included 30 patients with CRC; 30 patients other colorectal diseases (20 cases with irritable bowel disease and 10 cases with benign rectal polyps) as pathological control group and 30 healthy individuals as normal control group (employee and attendants without CRC) who were clinically, laboratory, and ultrasonographically free. All participants were Egyptians and of Egyptian descendants. Ethics was followed out in accordance with the Helsinki Declaration. An informed written consent was obtained from each individual and approval from local ethical committee was obtained.

Patients were enrolled in the study when they had one or more of the following clinical symptoms: bleeding per rectum, diarrhea, constipation, alternating bowel habit, abdominal pain, flatulence, presence of mucous in the stool, weight loss, and or anorexia. Patients with bleeding per‐rectum due to local causes, for example, piles, fissure, colonic obstruction, colonic perforation, suspected toxic megacolon, colectomy, other malignancy, and hepatic diseases (hepatitis, NASH, hepatobiliary cancer) were excluded from the study. However, positive hepatitis serology was not an exclusion criterion if no clinical signs of liver damage were present.

After interview questionnaire, complete physical and PR examination, blood sampling was taken at the time of diagnosis, no medical or surgical interference was done to the patients.

The following investigations were done to all included patients and normal controls:

1. Complete blood count on Sysmex CA1800 five differential part (Sysmex, Japan), prothrombin time (PT) on automated blood coagulation analyzer Sysmex c 660 (Sysmex, Japan), Liver, kidney function tests, and Carcinoembryonic antigen (CEA) were done on Cobas c 6000 (Roche Diagnostics, Mannheim, Germany).
2. Human golgi membrane protein 73 (GP73) was detected by immunoassay Kits from CUSABIO (Reader Aз 1851& Washer 909) from DAS (Italy), Catalog number CSB‐E11332h. All reagents, working standards and samples were prepared according to the manufacture's instructions. To each well,100 μl of standard and sample was added. Wells were covered with the adhesive strip provided and incubated for 2 h at 37°C. After removing the liquid of each well, 100 μl of Biotin‐antibody (1x) was added, covered with a new adhesive strip and incubated for 1 h at 37°C. Each well was aspirated and washed for three times using 200 μl of the wash buffer per wash. At each wash step, the sample was left for 2 min ensuring complete removal of liquid. To each well 100 μl of HRP‐avidin was added. The wells were covered with a new adhesive strip andincubated for 1 h at 37°C. As previously mentioned,the wash step was repeated. 90 μl TMB substrate was added to each well then incubated for 15–30 min at 37°C in dark. Followed by addition of stop solution to each well. Within 5 min, optical density of each well was determined, using a microplate reader set to 450 and 540 nm.

### 2.1 Statistical analysis of the data

Data were fed to the computer and analyzed using IBM SPSS software package version 20.0. Kolmogorov–Smirnov was used to verify the normality of distribution of variables; Comparisons between groups for categorical variables were assessed using Chi‐square test (Monte Carlo). Student *t* test was used to compare two groups for normally distributed quantitative variables while ANOVA was used for comparing the studied groups and followed by post hoc test (Tukey) for pairwise comparison. Kruskal–Wallis test was used to compare different groups for abnormally distributed quantitative variables and followed by post hoc test (Dunn's for multiple comparisons test) for pairwise comparison. Receiver operating characteristic curve (ROC) was used to determine the diagnostic performance of the markers, area more than 50% gives acceptable performance and area about 100% is the best performance for the test. Significance of the obtained results was obtained at the 5% level.

## 3 RESULTS

**FIGURE 1:**
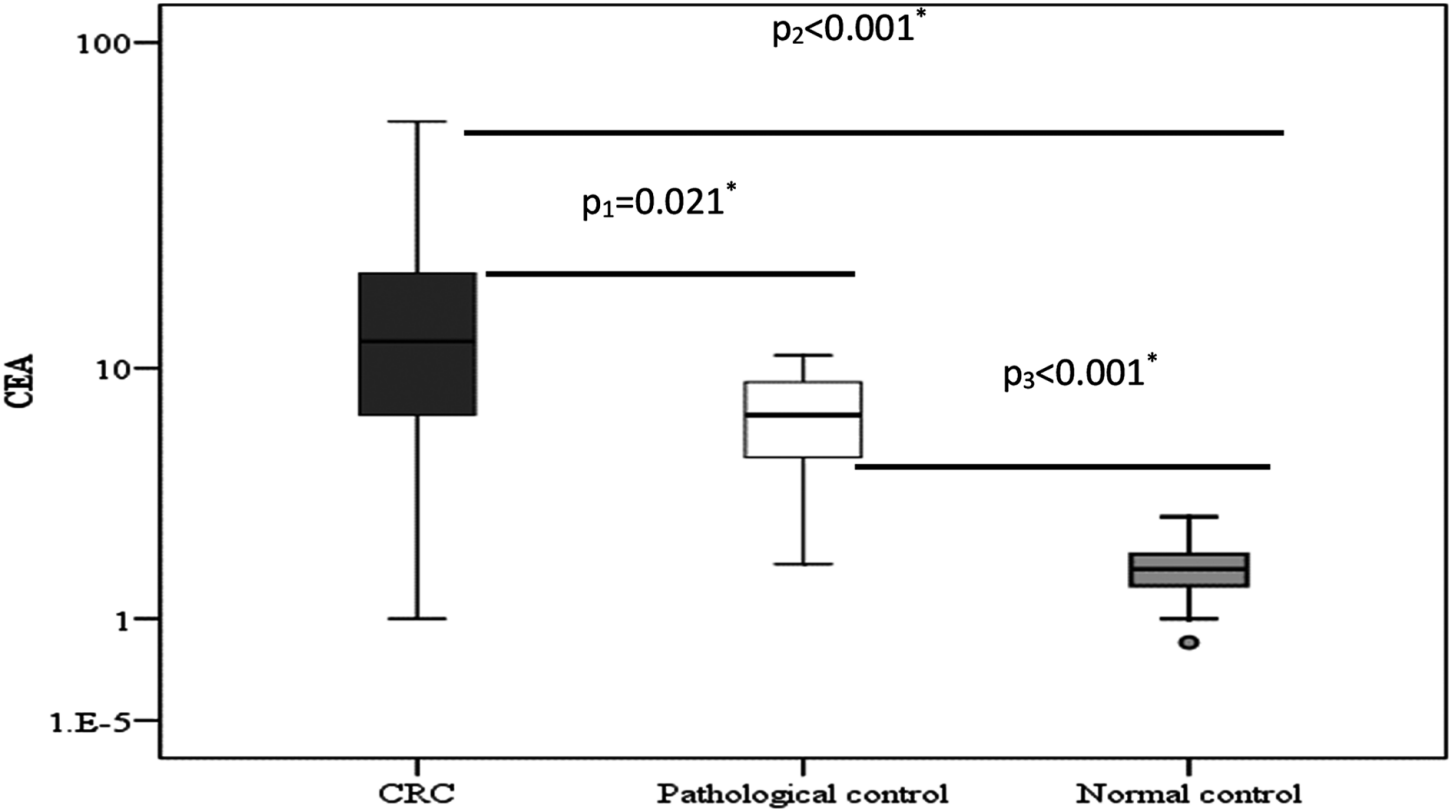
Boxplots of mean CEA levels in all studied groups (CRC vs. pathological and normal control)

**FIGURE 2:**
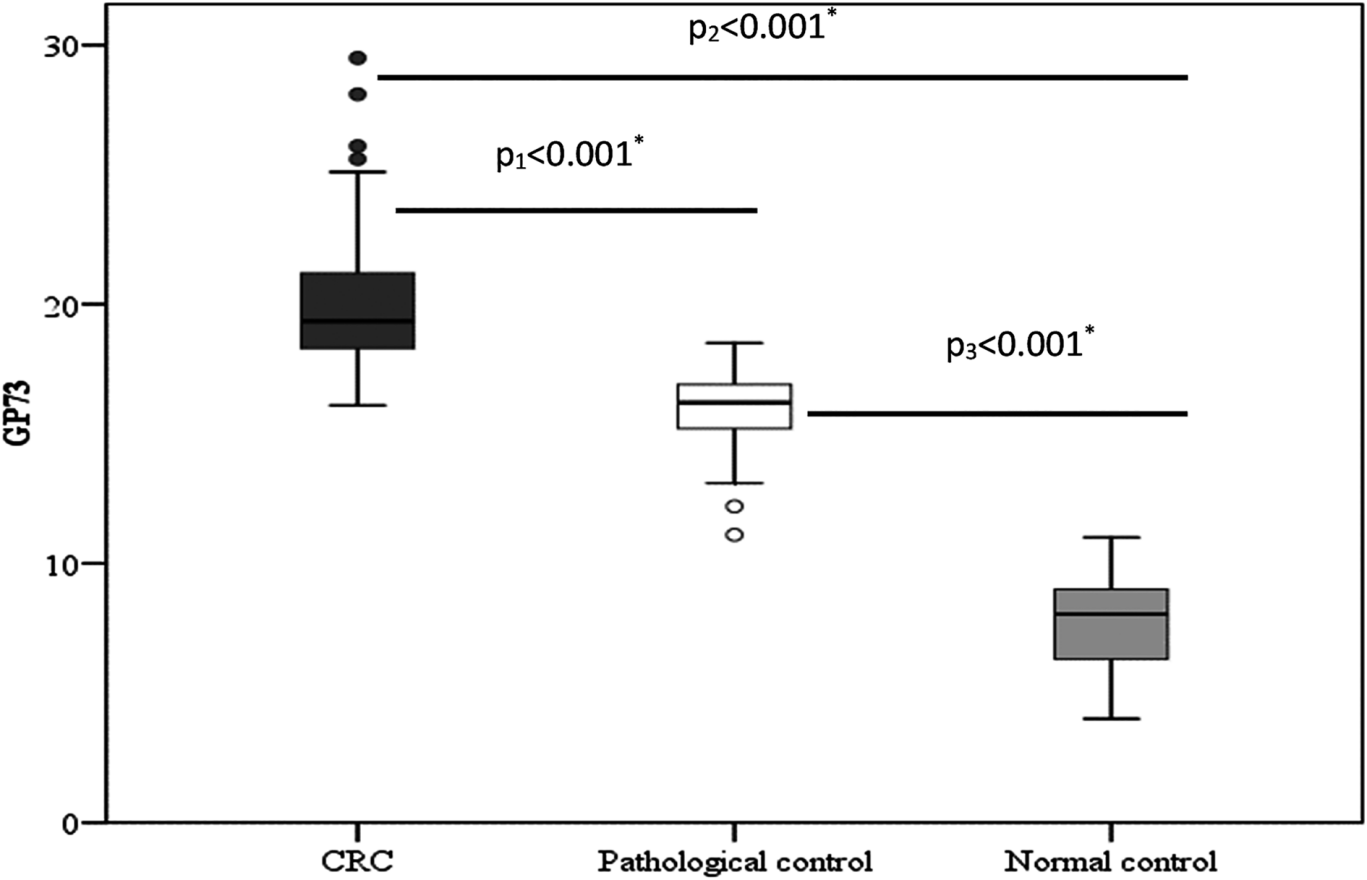
Boxplots of mean GP73 levels in all studied groups (CRC vs. pathological and normal control)

**FIGURE 3:**
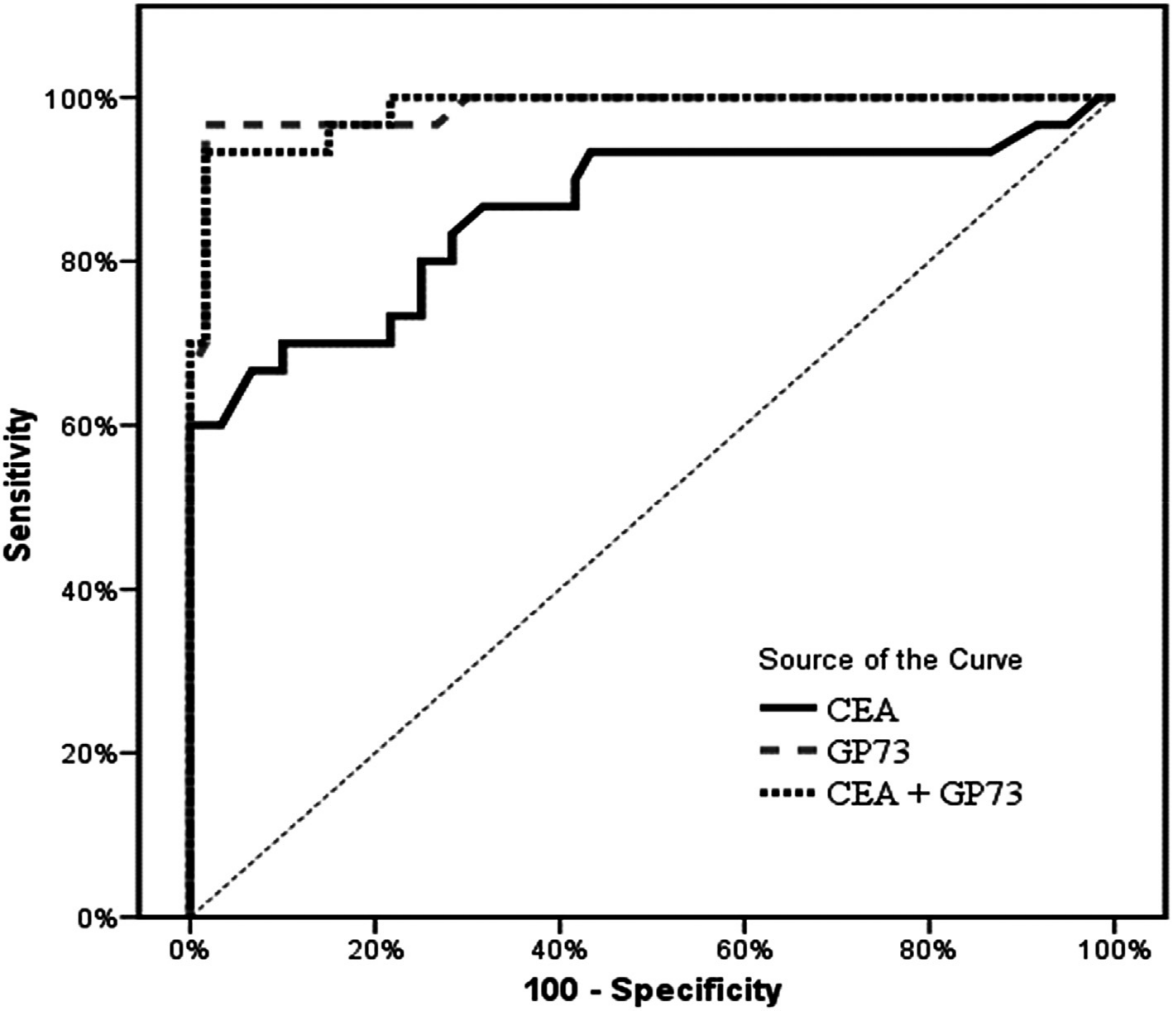
Receiver operating characteristic curve studying the validity of gp73, CEA, and combined marker of GP73 and CEA as diagnostic biomarkers for CRC

Table 1 showed the sociodemographic data and clinicopathological features of CRC group versus pathological and normal control groups, there was 21 males (70%) and 9 females (30%) in the CRC group, 15 of urban resident (50%) and 15 of rural resident (50%) and all the sociodemographic parameters are insignificant among groups (*p* > .05) except for sex and presence of diabetes mellitus. Clinical features of CRC were shown in Table 2 and nearly most of clinical symptoms are significant for CRC patients versus pathological and normal control groups; Hematological and biochemical tests of CRC patients versus pathological and normal control groups were almost insignificant except for GP73 and CEA levels are significant as shown in Tables 3 and 4 showed positive significant correlation between GP73 and CEA and no significant correlation with different other parameters in CRC group by spearman correlation coefficient test. Table 5 showed the diagnostic performance of GP73 & CEA for diagnosing colorectal carcinoma. The best cutoff value was >17.5 IU/ml in GP73 compared to >9 ng/ml with CEA. GP73 had the highest sensitivity and specificity as well as higher positive and negative predictive values compared to CEA alone or combined with GP73. Table 6; showed no statistical significant relation between gp73 level and sex, residence, and marital status, smoking, history of blood transfusion, presence of DM, hypertension, and lymph node metastasis *p* > .05. Figures 1 and 2 showed boxplots of CEA and GP73 levels in all studied groups. CEA were significantly higher in CRC patients compared to pathological and normal control groups (Figure 1; *p* < .021, .001, and .001, respectively). And GP73 levels were significantly higher in CRC patients compared to other groups (Figure 2; *p* < .001, .001, and .001, respectively). To assess the diagnostic value of GP73, CEA, and combined marker of both of them in diagnosis of CRC, we performed ROC curve analysis to differentiate patients with CRCI from non‐CRC patients (Figure 3). This indicated that gp73, CEA, and combined GP73 and CEA might have good diagnostic value for CRC (*p* value <.001).

**TABLE 1:** Sociodemographic and clinicopathological features of CRC versus pathological and normal control groups

	CRC(*n* = 30)	Control		Test of sig.	*p*
		Pathological(*n* = 30)	Normal(*n* = 30)		
Sex					
Male	21 (70%)	12 (40%)	15 (50%)	*χ* ^2^ = 24.911^a^	<.001^a^
Female	9 (30%)	18 (60%)	15 (50%)		
Age					
Mean ± SD	58 ± 7.3	56.9 ± 6.3	59.5 ± 7.5	*F* = 0.972	.382
Median (Min–Max)	57 (46–80)	57 (41–65)	61 (43–73)		
Marital status					
Married	26 (86.7%)	24 (80%)	22 (73.3%)	*χ* ^2^ = 4.833	^MC^ *p* = .537
Divorced	0 (0%)	1 (3.3%)	0 (0%)		
Single	4 (13.3%)	5 (16.7%)	7 (23.3%)		
Residence					
Urban	15 (50%)	20 (66.7%)	21 (70%)	*χ* ^2^ = 4.021	.134
Rural	15 (50%)	10 (33.3%)	9 (30%)		
			
Smoking					
Yes	9 (30%)	6 (20%)	2 (6.7%)	*χ* ^2^ = 5.36	.068
No	21 (70%)	24 (80%)	28 (93.3%)		
			
Blood transfusion					
Yes	10 (33.3%)	10 (33.3%)	6 (20%)	*χ* ^2^ = 1.731	.421
No	20 (66.7%)	20 (66.7%)	24 (80%)		
DM					
Yes	5 (16.7%)	14 (46.7%)	15 (50%)	*χ* ^2^ = 8.603^a^	.014^a^
No	25 (83.3%)	16 (53.3%)	15 (50%)		
HTN					
Yes	8 (26.7%)	10 (33.3%)	11 (36.7%)	*χ* ^2^ = 0.712	.700
No	22 (73.3%)	20 (66.7%)	19 (63.3%)		
Contraceptives					
Yes	0 (0%)	0 (0%)	0 (0%)	‐	‐
No	9 (100%)	18(100%)	15 (100%)		
CRC staging					
I	15	‐	‐		
II	10	‐	‐	‐	‐
III	5	‐	‐		
IV	0	‐	‐		
Tumor size (cm)					
<3	20	‐	‐		
3–5	10	‐	‐	‐	‐
LN involvement					
Yes	10	‐	‐	‐	‐
No	20	‐	‐		
Distant metastasis					
Yes	0	‐	‐	‐	‐
No	30	‐	‐		

*Note: χ* ^2^, Chi square test; MC, Monte Carlo; FE, Fisher exact; *F*, *F* for ANOVA test; *p*, *p* value for comparing between the studied groups; DM, diabetes mellitus; HTN, hypertension; LN, lymph node.

^a^ Statistically significant at *p* ≤ .05.

**TABLE 2:** Clinical features of CRC patients versus pathological and normal control groups

	CRC(*n* = 30)	Control		*χ* ^2^	*p*
		Pathological(*n* = 30)	Normal(*n* = 30)		
Loss of weight					
Yes	14 (46.7%)	6 (20%)	0(0%)	8.086^a^	.018^a^
No	16 (53.3%)	24 (80%)	30 (100%)		
Loss of appetite					
Yes	17 (56.7%)	9 (30%)	0 (0%)	8.038^a^	.018^a^
No	13 (43.3%)	21 (70%)	30 (100%)		
Fever					
Yes	4 (13.3%)	5 (16.7%)	0 (0%)	0.635	^MC^ *p* = .942
No	26 (86.7%)	25 (83.3%)	30 (100%)		
Diarrhea					
Yes	1 (3.3%)	5 (16.7%)	0 (0%)	2.848	^MC^ *p* = 0.248
No	29 (96.7%)	25 (83.3%)	30 (100%)		
Abd pain					
Yes	13 (43.3%)	17 (56.7%)	8 (26.7%)		
No	17 (56.7%)	13 (43.3%)	22 (73.3%)	5.557	.062
Bleeding rectum					
Yes	14 (46.7%)	20 (66.7%)	0(0%)	20.469^a^	<.001^a^
No	16 (53.3%)	10 (33.3%)	30 (100%)		
Constipation					
Yes	7 (23.3%)	6 (20%)	3 (10%)	0.373	.830
No	23 (76.7%)	24 (80%)	22 (90%)		
Easy fatigability					
Yes	13 (43.3%)	21 (70%)	0 (0%)	17.376^a^	<.001^a^
No	17 (56.7%)	9 (30%)	30 (100%)		
Nausea					
Yes	5 (16.7%)	3 (10%)	3 (10%)	0.833	^MC^ *p* = .780
No	25 (83.3%)	27 (90%)	27 (90%)		
Vomting					
Yes	0 (0%)	4 (13.3%)	2 (6.7%)	4.086	^MC^ *p* = .157
No	30 (100%)	26 (86.7%)	28 (93.3%)		
Dyspepsia					
Yes	8 (26.7%)	14 (46.7%)	5 (16.7%)	6.667^a^	.036^a^
No	22 (73.3%)	16 (53.3%)	25 (83.3%)		
Abd distention					
Yes	7 (23.3%)	10 (33.3%)	6 (20%)	1.518	.468
No	23 (76.7%)	20 (66.7%)	24 (80%)		
Cachexia					
Yes	9 (30%)	8 (26.7%)	0 (0%)	10.588^a^	.005^a^
No	21 (70%)	22 (73.3%)	30 (100%)		
Pallor					
Yes	4 (13.3%)	7 (23.3%)	0 (0%)	8.314^a^	^MC^ *p* = .014^a^
No	26 (86.7%)	23 (76.7%)	30 (100%)		

*Note: χ* ^2^: Chi square test; MC, Monte Carlo; *p*, *p* value for comparing between the studied groups.

^a^ Statistically significant at *p* ≤ .05.

**TABLE 3:** Hematological and biochemical parameters among the studied population

	CRC(*n* = 30)	Control			*p*
		Pathological(*n* = 30)	Normal(*n* = 30)		
Total Bilirubin mg/dl					
Mean ± SD	1.2 ± 1.0	08 ± 0.5	1.02 ± 0.36		.15
Median (Min–Max)	1.1 (0.40–2.4)	0.6 (0.70–1.3)	1 (0.40–1.8)		
Sig. bet. grps.	*p*_1_ > .1^a^, *p*_2_ = .183, *p*_3_ > .1^a^				
Direct bilirubin mg/dl					
Mean ± SD	1.1 ± 0.4	0.2 ± 0.1	0.47 ± 0.21		.25
Median (Min–Max.)	0.50 (0.10–1.5)	0.1 (0.1–0.3)	0.45 (0.20–1.2)		
Sig. bet. grps.	*p*_1_ > .1^a^, *p*_2_ = .355, *p*_3_ > .1^a^				
Indirect bilirubin mg/dl					
Mean ± SD	0.76 ± 0.53	0.6 ± 0.27	0.46 ± 0.28		.35
Median (Min–Max)	0.60 (0.20–0.6)	0.7 (0.050–0.2)	0.35 (0.20–0.5)		
Sig. bet. grps.	*p*_1_ > .1^a^, *p*_2_ = .251, *p*_3_ > .1^a^				
S. ALB g/dl					
Mean ± SD	3.9 ± 0.70	4.6 ± 0.44	4.1 ± 0.36		.5
Median (Min–Max.)	3.5 (3.1–4.7)	3.6 (3.7–4.9)	4 (3.5–4.8)		
Sig. bet. grps.	*p*_1_ > .1^a^, *p*_2_ > .1^a^, *p*_3_ > .1^a^				
AST U/l					
Mean ± SD	35.0 ± 22	32.4 ± 28	23.5 ± 24.7		.14
Median (Min–Max)	31.5 (24–54)	29 (23–59)	24 (17–48)		
Sig. bet. grps.	*p*_1_ = .092, *p*_2_ = .071^a^, *p*_3_ = .110				
ALT U/l					
Mean ± SD	25 ± 19.5	32.4 ± 16.5	19.4 ± 9.2		.6
Median (Min–Max)	24 (20–53)	38.5 (19–49)	33 (15–37)		
Sig. bet. grps.	*p*_1_ = .77, *p*_2_ = .8^a^, *p*_3_ = .367				
PT/sec					
Mean ± SD	13.6 ± 2.3	12.8 ± 2.4	12.5 ± 1.02		.75
Median (Min–Max)	13.2 (11.6–13.9)	12.8 (12.8–14)	12.3 (11.6–13.0)		
Sig. bet. grps.	*p*_1_ > .1^a^, *p*_2_ = .90, *p*_3_ > .1^a^				
INR					
Mean ± SD	1.2 ± 0.34	1.17 ± 0.28	1.1 ± 0.10		.15
Median (Min–Max)	1.2 (1–1.3)	1.7 (1.1–1.3)	1 (1–1.3)		
Sig. bet. grps.	*p*_1_ > .1^a^, *p*_2_ = .23^a^, *p*_3_ > .21^a^				
HB g/dl					
Mean ± SD	12.4 ± 1.3	10.1 ± 1.1	12.2 ± 1.3	*F* = 34.059^a^	<.01^a^
Median (Min–Max)	12.4 (10.2–15)	10 (7.5–12.3)	12 (9.8–15)		
Sig. bet. grps.	*p*_1_ < .01^a^, *p*_2_ = .797, *p*_3_ < .01^a^				
WBCs x 10^3^/ul					
Mean ± SD	5.1 ± 2.1	4 ± 1.3	6.5 ± 2.1	*H* = 21.733^a^	<.01^a^
Median (Min–Max)	4.8 (2.3–10.5)	3.9 (1.7–7.2)	6.2 (3.5–10.5)		
Sig. bet. grps.	*p*_1_ = .029^a^, *p*_2_ = .013^a^, *p*_3_ < .01^a^				
Platelets x10^6^/ul					
Mean ± SD	180.0 ± 55.2	175.03 ± 43.7	244.8 ± 76.3	*H* = 14.102^a^	<.01^a^
Median (Min–Max)	196 (150–252.0)	172 (140–221)	222 (145–410)		
Sig. bet. grps.	*p*_1_ = .104, *p*_2_ < .01^a^, *p*_3_ < .01^a^				
S. Creatinine mg/dl					
Mean ± SD	0.97 ± 0.35	1.1 ± 0.43	0.82 ± 0.20	*H* = 8.682^a^	.013^a^
Median (Min–Max)	0.90 (0.50–2.2)	1.1 (0.40–2.1)	0.80 (0.40–1.2)		
Sig. bet. grps.	*p*_1_ = .159, *p*_2_ = .125, *p*_3_ = .003^a^				
HCVab					
Yes	3(10%)	1 (3.3%)	0 (0%)	*χ* ^2^ = 3.025	^MC^ *p* = .318
No	27 (90%)	29 (96.7%)	30 (100%)		
HBsAg					
Yes	2 (6.7%)	1 (3.3%)	0 (0%)	*χ* ^2^ = 1.886	^MC^ *p* = .770
No	28 (93.3%)	29 (96.7%)	30 (100%)		
CEA ng/ml					
Mean ± SD	58.0 ± 17.7	6.8 ± 2.6	2.3 ± 0.8	*H* = 55.555^a^	<.001^a^
Median (Min–Max)	42.2 (11–88)	7 (1.9–11)	1.8 (0.70–3)		
Sig. bet. grps.	*p*_1_ = .021^a^, *p*_2_ < .001^a^, *p*_3_ < .001^a^				
GP73 IU/ml					
Mean ± SD	20.7 ± 3.4	15.9 ± 1.6	5.0 ± 1.4	*F* = 217.391^a^	<.001^a^
Median (Min–Max)	19.4 (16.1–29.5)	16.2 (11.1–18.5)	4.1 (4–6)		
Sig. bet. grps.	*p*_1_ < .001^a^, *p*_2_ < .001, *p*_3_ < .001^a^				

*Note: χ* ^2^, Chi square test. MC, Monte Carlo. *F*, *F* for ANOVA test, pairwise comparison bet. Each two groups was done using post hoc test (Tukey). *H*, *H* for Kruskal–Wallis test, pairwise comparison bet. Each two groups was done using post hoc test (Dunn's for multiple comparisons test). *p*, *p* value for comparing between the studied groups. *p*_1_, *p* value for comparing between CRC and Pathological control. *p*_2_, *p* value for comparing between CRC and normal control. *p*_3_, *p* value for comparing between pathological and normal control. Sig. bet. grps., significance between groups. S. ALB, serum albumin. AST, aspartate aminotransferase. ALT, alanine aminotransferase. PT, prothrombin time INR:International normalized ratio. HB, hemoglobin level. WBCs, white blood cells. HCVab, hepatitis C virus antibodies; HBsAg, hepatitis B surface antigen. CEA, carcenoembryonic antigen. Gp73, golgi protein 73.

^a^ Statistically significant at *p* ≤ .05.

**TABLE 4:** Correlation between GP73 and different parameters in CRC group (*n* = 30)

	GP73	
	*r*_s_	*p*
Age (years)	−0.018	.925
Direct bilirubin	−0.015	.937
Indirect bilirubin	0.014	.940
S. ALB	−0.115	.544
AST	0.065	.733
ALT	−0.094	.620
PT	−0.247	.189
INR	0.027	.887
HB	0.045	.814
WBCs	−0.417	0.052
Platelets	−0.111	.561
S. Creatinine	0.190	.314
Tumor size	0.040	.08
CEA	0.455^a^	.011^a^

*Note: r*_s_, Spearman coefficient.

^a^ Statistically significant at *p* ≤ .05.

**TABLE 5:** Agreement (sensitivity, specificity) for diagnosis of CRC patients (*n* = 30) from pathological and normal controls (*n* = 60)

	AUC	*p*	95% CI	Cut off	Sensitivity	Specificity	PPV	NPV
CEA	0.860	<.001^a^	0.766–0.954	>9	70.0	90.0	77.8	85.7
GP73	0.986	<.001^a^	0.964–1.007	>17.5	96.67	98.33	96.7	98.3
CEA + GP73	0.984	<.001^a^	0.963–1.007	–	93.33	98.33	96.6	96.7

Abbreviations: AUC, area under a curve; CI, confidence intervals; NPV, negative predictive value; *p* value, probability value; PPV, positive predictive value.

^a^ Statistically significant at *p* ≤ .05.

**TABLE 6:** Relation between GP73 and different parameters in CRC group (*n* = 30)

	GP73		Test of sig.	*p*
	Min–Max	Mean ± SD		
Sex				
Male	19.5 (16.1–29.5)	20.6 ± 3.3	*t* = 0.072	.493
Female	19.1 (18.2‐28.1)	20.8 ± 4.1		
Residence				
Urban	19.75 (18.0‐29.5)	21.5 ± 3.9	*t* = 1.333	.193
rural	19.05 (16.1‐26.1)	19.9 ± 2.7		
Marital status				
Married	20 (18.0–29.5)	21.2 ± 4.2	*F* = 0.348	.791
Divorced	19.65 (18.1‐25.0)	20.2 ± 2.5		
Single	18.3 (18.2‐20.5)	19.0 ± 1.3		
Smoking				
Yes	20 (18.1‐29.5)	21.6 ± 4.3	*t* = 0.993	.329
No	19.2 (16.1‐28.1)	20.3 ± 2.9		
Contraceptives				
Yes	18.7 (16.1–29.5)	20.2 ± 3.5	*t* = 0.895	.379
No	20.05 (18.1‐28.1)	21.3 ± 3.1		
Blood transfusion				
Yes	19.25 (18.1–29.5)	21.7 ± 4.3	*t* = 1.037	.319
No	19.35 (16.1–28.1)	20.1 ± 2.8		
DM				
Yes	19.5 (18.1–29.5)	22.9 ± 5.5	*t* = 1.066	.341
No	19.2 (16.1–26.1)	20.2 ± 2.7		
HTN				
Yes	19.35 (18.2‐29.5)	21.7 ± 4.0	*t* = 0.999	.326
No	19.35 (16.1–28.1)	20.3 ± 3.1		
Distant Metastasis				
Yes	b	b	b	b
No	19.4(16.1–29.5)	20.7 ± 3.4		
LN involvement				
Yes	19.35 (18.1–29.5)	21.6 ± 3.8	*t* = 1.351	.190
No	19.35 (16.1–28.1)	19.9 ± 2.8		

*Note: F*, ANOVA test; *p*: *p* value for association between different categories; *t*, Student *t* test.

^a^ Statistically significant at *p* ≤ .05.

^b^ Excluded from the association due to small number of case (*n* = 1).

## 4 DISCUSSION

Some factors interfere with the early diagnosis of CRC as most patients have no or nonspecific symptoms in the early stages CRC in addition to the presence of some defects in early diagnosis, determination of prognostic factors and metastatic disease treatment despite the great progress in the screening and management programs. So is mandatory to find a noninvasive, sensitive, specific, and cost‐effective test that helps the early diagnosis of CRC.^9^

Blood‐based CRC biomarkers should be easy to perform, not risky to the patient, can be repeated at shorter intervals, identify the high risk population therefore allowing early detection and reducing CRC incidence rate.^10^ Due to the heterogeneous nature of CRC, it difficult to find a single sensitive and specific screening biomarker for CRC. Instead, multiple markers may be combined to early detect CRC.^1^ Nikolaou et al stated that CRC servillance and monitoring treatment response Using CEA has asensitivity and specificity ranging from 40 to 70%. So, it is not convenient to CEA for screening or diagnosis of CRC.^11^

Tumor marker tests such as CEA are used most often along with other tests to monitor patients who have already been diagnosed with colorectal cancer. as tumor marker levels can sometimes be normal in someone who has cancer and can be abnormal for reasons other than cancer.

CEA levels were highly statistically different between the groups of the current study as shown in Table 3. In 2012, Nakatani et al^12^ provided that sigma region colon cancer had very high CEA. According to Vukobrat‐Bijedic et al, metastatic colon cancer especially to lymph nodes caused marked elevation of CEA with the highest level was in patients with in the lung and liver metastases (2098 ± 2727.4) while the lowest level was found in metastases to the nearby adipose tissue (1.66 ± 0) that was statistically insignificant (*p* > .05).Uncomplicated CRC patients usually have average values. Other studies revealed that there was no significant difference in CEA levels in CRC patients with lymph nodes metastases. Similarly, some reports found that 9.9% of patients with lymph node metastases did not have increased CEA levels. Extremely high CEA was found in the right hemicolon cancer patients.^13^

CEA is also used to monitor CRC recurrence. High CEA after tumor resection indicates poor prognosis and cancer progression. The sensitivity of CEA increases with tumor stage and decreases after tumor resection. CEA is not specific for CRC but can elevated in inflammatory bowel disease, pancreatitis, liver disease, or other malignancies. High CEA levels maybe found in advanced stages in some CRC patients. So, CEA cannot be used as effective screening tool.^14^ However, CEA remains a useful diagnostic test. Serum CEA has high specificity and low sensitivity during monitoring for CRC recurrence. The cut‐off for optimal sensitivity ranged from 3 to 15 and 2.2 ng/ml for optimal specificity. At this level, CEA may help in follow up after surgical treatment.^15^

In our study, comparing GP73 level between groups revealed a high statistical difference as being high in CRC group in comparison to pathological group and normal control group *p* < .001. In 2011, Ozal et al stated that colon cancer patients with liver metastases had high serum GP73.^8^ Others reported increased GP73 serum levels in Hcc and increased tissue levels in adenocarcinomas of prostate, colon, breast, and some types of renal cell carcinomas indicating thatGP73 is not a specific biomarker for HCC.^16^

Previous studies addressing the relation of Gp73 and CRC are few and we highlighted and focused on their results in relation to the results of the current study. The first one by Block et al included in their study human HCC serum samples from two sources for the analysis of GP73. HBV and HCC serum samples and sera from patients diagnosed CRC. Using immunoblot, they decleared that the elevation of GP73 was noticed with HCC and HBV‐induced HCC. They did not find elevated GP73 levels in patients with colorectal cancer. We excluded any patients with history of hepatic diseases or hepatic cancer from our study and GP73 was elevated in CRC patient although they do not have any liver affection indicating that GP73 serves a useful noninvasive sensitive and specific marker for early detection of CRC.^17^

Ozal et al included 126 patients with liver only metastases, with nonliver metastases, without distant metastasis (both preoperative and postoperative period), and a group of colon cancer patients in remission at least 3 years and not relapsed within at least 6 months after obtaining blood samples and a healthy group with similar age and gender in their study. In contrast, our study included 30 patients with CRC, 30 other colorectal diseases (20 patients with irritable bowel disease and 10 patients with benign rectal polyps) as pathological control and 30 healthy subjects as control (employee and attendants without CRC) who were clinically, laboratory and ultrasonographically free. They concluded that the diagnostic performance of gp73 as a tumor marker was especially more prominent in the subgroup of liver only metastases. For the non‐liver metastases, the performance of CEA was similar to gp73.^8^

Our results showed that GP73 had higher sensitivity and specificity as well as higher positive and negative predictive values than CEA concluding that GP73 serves a useful noninvasive sensitive and specific marker for early detection of CRC. And the third study by El‐Zefzafy et al^18^ studied serum Dickkopf‐1, and Golgi membrane protein in Egyptian patients with colorectal cancer as a diagnostic tool for colorectal cancer. They showed that serum Dickkopf‐1 was highly significant increase in its levels in patients with CRC and patients with other colorectal disease while no significant difference in GP73 between the studied groups was found. Although our study is similar to El‐Zefzafy et al, we only studied GP73 and in contrast to their result, we found that GP73 is a useful liquid tumor marker for CRC cases with high sensitivity and specificity.

Comparing the diagnostic performance GP73 against that of CEA in patients with CRC, GP73, had a better diagnostic performance than CEA. We found that at cut‐off level of >17.5 IU/ml, GP73 sensitivity of was 96.8% and its specificity was 98.3%. while at the level of >9 ng/ml, the sensitivity and specificity of CEA were 70 and 90%. We concluded that the cut off value, sensitivity and specifity, positive, and the negative predictive value were higher in GP73 than CEA in CRC group.

According to Ozal et al, GP73 showed AUC = 0.974 ± 0.003 [95% CI: 0.912–1.037] in contrast to CEA which showed AUC = 0.859 ± 0.089 [95% CI: 0.684–1.034]. In CRC patients with liver metastasis, the GP73 diagnostic performance was better than CEA. On the contrary, in absence of liver metastases, the performance of CEA was comparable to gp73. At the cut‐off level of 15 ng/ml, GP73 sensitivity of was 80% and its specificity was 100%. At the level of 5 ng/ml, the sensitivity and specificity of CEA were 72 and 100%, respectively. Accordingly, serum gp73 seems to be a useful tumor marker in CRC patients.^8^

El‐Zefzafy et al in 2015 did not find a correlation between GP73 levels and CEA.^18^ On the opposite hand Ozal et al found the performance of CEA is similar to GP73 at the cut‐off level of 15 ng/ml.^8^

In the present study, there is a positive correlation between gp 73 and CEA level and gp 73 in patients with CRC by using spearman correlation coefficient test *p* < .05 and absence of correlation with other parameters. On the opposite hand El‐Zefzafy et al did not find a correlation between GP73 levels and CEA.^17^

The main limitation of this study was the small sample size, limited duration of the study, absence of patients with distant metastasis were included in the current study to explore the level of Gp73 in advanced stage of the disease.

## 5 CONCLUSIONS

GP73 has good diagnositic performance in CRC patients; Further studies on large scale of CRC patients with different stages of the disease are warranted to elucidate its clinical value in CRC patients.

## CONFLICT OF INTEREST

Authors declare there is no conflict of interest.

## AUTHOR CONTRIBUTIONS

**Shaymaa Abdelraheem:** Methodology; project administration; resources; visualization; writing‐original draft; writing‐review & editing. **Heba Attya:** Investigation; project administration; visualization; writing‐original draft; writing‐review & editing. **Mohamed Abdo:** Investigation; methodology; resources; supervision. **Fadia Attia:** Conceptualization; supervision; validation; writing‐review & editing.

## ETHICS STATEMENT

Ethics was followed out in accordance with the Helsinki Declaration. An informed written consent was obtained from each individual and approval from local ethical committee at Suez Canal University, Faculty of Medicine.

## Data Availability

The data that support the findings of this study are available from the corresponding author upon reasonable request. The data are not publicly available due to privacy or ethical restriction.
